# Carbon ion reirradiation compared to intensity-modulated re-radiotherapy for recurrent head and neck cancer (CARE): a randomized controlled trial

**DOI:** 10.1186/s13014-020-01625-0

**Published:** 2020-08-05

**Authors:** Thomas Held, Kristin Lang, Sebastian Regnery, Katharina Weusthof, Adriane Hommertgen, Cornelia Jäkel, Eric Tonndorf-Martini, Johannes Krisam, Peter Plinkert, Karim Zaoui, Christian Freudlsperger, Julius Moratin, Jürgen Krauss, Semi B. Harrabi, Klaus Herfarth, Jürgen Debus, Sebastian Adeberg

**Affiliations:** 1 Department of Radiation Oncology, Heidelberg University Hospital, Im Neuenheimer Feld 400, 69120 Heidelberg, Germany; 2 Heidelberg Institute of Radiation Oncology (HIRO), Heidelberg, Germany; 3 National Center for Tumor Diseases (NCT), Heidelberg, Germany; 4 Institute of Medical Biometry and Informatics (IMBI), Heidelberg University Hospital, Heidelberg, Germany; 5 Department of Otorhinolaryngology, University of Heidelberg, Heidelberg, Germany; 6 Department of Oral and Maxillofacial Surgery, University Hospital Heidelberg, Heidelberg, Germany; 7 Department of Medical Oncology, National Center for Tumor Diseases (NCT), Heidelberg University Hospital, Heidelberg, Germany; 8 Clinical Cooperation Unit Radiation Oncology, German Cancer Research Center (DKFZ), Heidelberg, Germany; 9 Heidelberg Ion-Beam Therapy Center (HIT), Heidelberg, Germany; 10 German Cancer Consortium (DKTK), partner site Heidelberg, German Cancer Research Center (DKFZ), Heidelberg, Germany

**Keywords:** Head and neck cancer, Reirradiation, Toxicity, Local control, Particle therapy, Heavy ions, Squamous cell carcinoma, Intensity-modulated radiotherapy

## Abstract

**Background:**

Intensity-modulated re-radiotherapy (reIMRT) has been established as a standard local treatment option in patients with non-resectable, recurrent head and neck cancer (rHNC). However, the clinical outcome is unfavorable and severe toxicities (≥grade III) occurred in 30–40% of patients. The primary aim of the current trial is to investigate carbon ion reirradiation (reCIRT) compared to reIMRT in patients with rHNC regarding safety/toxicity as well as local control, overall survival (OS), and quality of life (QoL).

**Methods:**

The present trial will be performed as a single center, two-armed, prospective phase II study. A maximum of 72 patients will be treated with either reIMRT or reCIRT to evaluate severe (≥grade III) treatment-related toxicities (randomization ratio 1:1). The primary target value is to generate less than 35% acute/subacute severe toxicity (≥grade III), according to the Common Terminology Criteria for Adverse Events v5.0, within 6 months after study treatment. The total dose of reirradiation will range between 51 and 60 Gy or Gy (RBE), depending primarily on the radiotherapy interval and the cumulative dose to organs at risk. Individual dose prescription will be at the discretion of the treating radiation oncologist. The local and distant progression-free survival 12 months after reirradiation, the OS, and the QoL are the secondary endpoints of the trial. Explorative trial objectives are the longitudinal investigation of clinical patient-related parameters, tumor parameters on radiological imaging, and blood-based tumor analytics.

**Discussion:**

Recent retrospective studies suggested that reCIRT could represent a feasible and effective treatment modality for rHNC. This current randomized prospective trial is the first to investigate the toxicity and clinical outcome of reCIRT compared to reIMRT in patients with rHNC.

**Trial registration:**

ClinicalTrials.gov; NCT04185974; December 4th 2019.

## Background

Around 30–50% of patients with locally advanced head and neck cancer (HNC) develop local recurrence or tumor progression after initial multimodal therapy [1, 2]. Local tumor growth can lead to severe symptoms such as dysphagia, cachexia, and tumor pain with a significant decrease of the quality of life (QoL). The therapeutic options are limited in this highly pre-treated, vulnerable patient cohort. Depending on the tumor localization, salvage surgery should be evaluated in patients with good performance status with a 2-year progression-free survival around 70% for stage I/II and 30% for stage III rHNC [3]. However, only a subset of patients (approximately 30–50%) is suitable for a salvage surgery in the clinical routine. Palliative systemic therapies showed limited positive effects with the “EXTREME” regimen resulting in overall survival (OS) rates of 10.1 months and around 80% severe treatment toxicity (≥grade III) [4]. Recently, immunotherapy has emerged as a treatment option for patients with recurrent or metastatic head and neck squamous cell carcinoma (HNSCC) [5].

The clinical routine for reirradiation of rHNC is reIMRT [6, 7]. Definitive reIMRT is commonly applied with a total dose up to 60 Gy in 2 Gy fractions [8]. In this heavily pretreated patient cohort, reIMRT was associated with unfavorable rates of treatment-related toxicity (≥grade III) in 30–40% of patients [8–10]. The combination treatment of chemotherapy with photon reirradiation further intensified severe toxicities [6, 7] but showed unfavorable 2-year OS rates ranging from 15 to 26% in patients with rHNC. Stereotactic body radiation therapy (SBRT) has emerged as a viable therapeutic option for non-resectable rHNC with the potential to significantly reduce acute and chronic treatment associated toxicity [11, 12]. However, the clinical benefit of reirradiation with SBRT compared to reIMRT has yet to be demonstrated in a prospective clinical trial. High total doses from previous irradiation treatment generally limit the dose of reirradiation. The relative biological effectiveness (RBE) of carbon ions is higher compared to photons and it is associated with favorable effects in radioresistant tumors [13]. Because of the steep dose gradient, reCIRT is superior in sparing normal tissue and organs at risk, compared to reirradiation with photons. Consequently, carbon ions possibly represent a feasible and effective treatment modality for salvage reirradiation. A retrospective analysis on 229 consecutive patients with rHNC treated at our clinic with reCIRT showed encouraging results with 3.1% acute and 14.5% late severe (≥grade III) toxicity [14]. A median total dose of 51 Gy (RBE) in 17 fractions of 3 Gy (RBE) was safe and effective for reCIRT with a median OS of 13.7 months for patients with recurrent HNSCC. Data on reCIRT in patients with rHNC is rare, especially in the prospective setting. In the current CARE trial, the impact of reCIRT or reIMRT will be evaluated and the toxicity/safety and efficacy will be compared.

## Methods/design

**Fig. 1:**
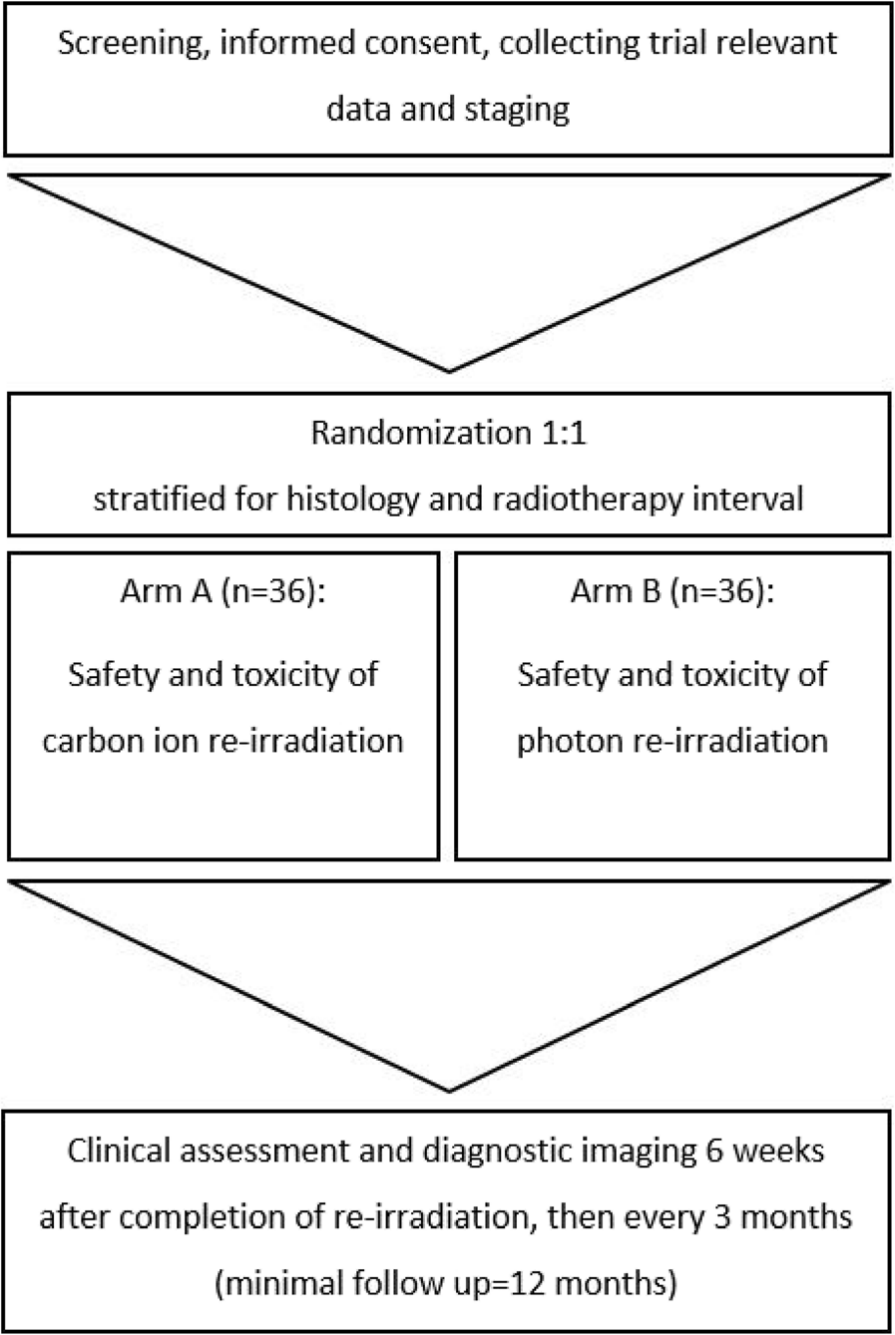
Study flow chart for trial subjects. A total of 72 patients will be randomized (1:1) to the experimental arm A (carbon ions) or the control arm B (photons)

The trial will be performed as a single-center, two-armed, randomized controlled phase II study. A maximum of 72 patients are projected to be enrolled into the study. Patients fulfilling the inclusion criteria will be treated with either reIMRT or reCIRT to evaluate severe (≥grade III) acute and subacute treatment-related toxicities (randomization ratio 1:1). Randomization will be stratified with respect to histology (HNSCC vs. others) and radiotherapy (RT) interval (≥2 years vs. < 2 years). Block randomization with varying block lengths will be performed. The overall duration of the trial is scheduled to be 60 months, consisting of 48 months of recruitment and a minimum follow-up of 12 months. A flow chart for trial subjects is shown in Fig. 1.

### Inclusion criteria

Inclusion criteria are as follows: Locally recurrent/progressive head and neck cancer after initial radiation therapy; microscopic or macroscopic tumor after salvage surgery; indication for reirradiation; completed wound healing after surgical intervention; Karnofsky Performance Score ≥ 60; age ≥ 18 years; written informed consent; ability of subject to understand character and individual consequences of the trial; for women of childbearing potential (and men), adequate contraception; and submission of previous RT records.

### Exclusion criteria

The exclusion criteria are as follows: Reirradiation of malignancy in the larynx; diagnosed plasymocytoma, sarcoma, or chordoma; previous reirradiation in-field; time interval < 6 months after initial RT; distant metastases (except pulmonary metastases); patients who have not recovered from acute toxicities of previous therapies; refusal of the patients to take part in the study; pregnant or lactating women; known carcinoma < 5 years ago (excluding carcinoma in situ of the cervix, basal cell carcinoma, squamous cell carcinoma of the skin) requiring immediate treatment that would interfere with the study therapy; and participation in another clinical study or observation period in a competing trial.

### Radiation therapy

Intensity controlled active raster scanning technology will be used for the application of reCIRT under image guidance with orthogonal X-rays and a daily position correction. A total of 17–20 fractions of 3 Gy (RBE) will be applied on 5–6 days per week, resulting in a total dose of reCIRT ranging between 51 and 60 Gy (RBE). According to standard procedures at our clinic, reIMRT will be performed under image guidance with daily computed tomography (CT) imaging and position correction. A total of 27–30 fractions of 2 Gy will be applied 5 days per week over a period of approximately 5–6 weeks using volume modulated arc therapy (VMAT). Elective nodal irradiation, which is associated with greater risk of acute toxicity, will not be performed.

#### Treatment planning

For reirradiation, patients will be immobilized using an individual immobilization mask. All patients will receive a non-contrast planning CT scan in 3 mm layer thickness and if possible, also contrast-enhanced CT or magnetic resonance imaging (MRI) for optimal target volume definition. Treatment planning will be conducted using the planning software Syngo PT-Planning (Siemens, Erlangen, Germany) including a biologic plan optimization for photon plans and Masterplan Oncentra MasterPlan® (Nucletron, Columbia, SC, USA), RayStation® (RaySearch Laboratories, Stockholm, Sweden) or Accuray Precision® Treatment Planning (Accuray, Sunnyvale, CA, USA) for photon plans. If available, the initial treatment plans will be imported in the planning software. A sum plan of the initial dose distribution and the reirradiation plan will be generated.

#### Dose prescription

The total dose of reirradiation will range between 51 and 60 Gy or Gy (RBE) based on clinical experience in reCIRT of rHNC at our institution within the last 10 years [14]. Patients with an RT interval of less than 2 years will receive 51 Gy (RBE) reCIRT in 3 Gy (RBE) or 54 Gy photons in 2 Gy. Patients with an RT interval of over 2 years can receive up to 60 Gy (RBE) reCIRT in 3 Gy (RBE) or 60 Gy photons in 2 Gy. Additional factors influencing the dose prescription include the total dose of the initial course of RT, the patient’s performance status, initial tumor localization, the tumor volume to be treated with reirradiation, and previous salvage surgery. Individual dose prescription will be at the discretion of the treating radiation oncologist. The maximum total dose of reirradiation for selected patients equals 60 Gy or Gy (RBE). Details on the dose specifications are summarized in Table 1.

**Table 1:** Dose specification for reIMRT and reCIRT

	CTV reIMRT	CTV reCIRT
Dose per fraction	2 Gy	3 Gy (RBE)
Total dose	54–60 Gy	51–60 Gy (RBE)
BED2Gy^a^	/	64–75 Gy

*Abbreviations*: *reIMRT* intensity-modulated re-radiotherapy, *reCIRT* carbon ion reirradiation, *CTV* clinical target volume, *RBE* relative biological effectiveness, *BED2Gy* biological effective dose in 2 Gy fractions, ^a^calculated according to the local effect model (LEM I) with an assumed alpha/beta of 2

The total dose will be prescribed to the maximum of the calculated dose distribution for the target volume. Treatment planning aims in the coverage of the clinical target volume (CTV) by the 95%-isodose-line for reCIRT and 90% coverage of the planning target volume (PTV) for photon plans. The relative biological effective dose of reCIRT will be calculated from the physical dose considering the local effect model LEM1 [13]. BED2Gy refers to the equivalent dose in conventional fractionation with 2 Gy fractions. As a precaution, the α/β value is uniformly set at 2 Gy, declaring the focus on organs at risk and the prevention of severe treatment-related toxicity. Target volumes of reCIRT will be defined as follows:

##### Gross tumor volume (GTV)

Tumor disease on planning CT scan, contrast enhanced CT scan or T1-weighted MRI.

##### Clinical target volume (CTV)

Adding 2–5 mm margin to the GTV including the resection cavity.

##### Plan target volume (PTV)

Adding 2–3 mm margin to the CTV depending on patient positioning and beam angles.

#### Organs at risk

Organs at risk such as the brain stem (α/β = 2), the optic system (α/β = 3), and the spinal cord (α/β = 2) will be contoured in accordance with clinical standards at our institution. Fractionation effects of particle therapy will be considered for cumulative dose calculation for organs at risk. Dose constraints for normal tissue and organs at risk in patients with an RT interval of less than 2 years will be respected according to Quantitative Analyses of Normal Tissue Effects in the Clinic (QUANTEC) [15, 16]. Approximate values for symptomatic necrosis of the brain, brainstem, spinal cord myelopathy, and optic neuropathy are listed as less than 3, 5, 1, and 3%, respectively, after RT [15]. Data on the tolerance of normal tissue and organs at risk for reirradiation are rare. Preclinical studies on reirradiation of rhesus monkeys suggest substantial recovery of the cervical spinal cord within 2 years after the initial course of RT [17]. At our institution, an increase of the dose tolerance of organs at risk in the second course of irradiation by 20% after 2 years has shown acceptable safety and treatment-associated toxicity in retrospective analyses [14]. Dose constraints of organs at risk will be closely guided by the clinical experience at our institution. The corresponding dose volume histograms for the organs at risk should not exceed the proposed values shown in Table 2.

**Table 2:** Proposed radiation tolerance of organs at risk for reirradiation

	Maximum cumulative BED2Gy (RT interval < 2 years)	Maximum cumulative BED2Gy (RT interval ≥ 2 years)	Comment
Brain stem *(α/β = 2)*	60	78 (**≙** + 30%)	Maximum (surface)
Optic chiasm *(α/β = 3)*	54	64.8 (**≙** + 20%)	Maximum
Optic nerves *(α/β = 3)*	54	64.8 (**≙** + 20%)	Maximum
Spinal cord *(α/β = 2)*	45	54 (**≙** + 20%)	Maximum
Other organs at risk	ALARA*		/

*Abbreviations*: *BED2Gy* biological effective dose in 2 Gy fractions, *RT* radiotherapy, *ALARA* as low as reasonably achievable

In some cases, sparing of certain organs at risk (e.g. the ipsilateral optic nerve) is not feasible because of direct tumor infiltration. If neurological deficits are expected, because adherence to maximum dose constraints in the respective organs at risk is not reasonably achievable, the patient will be informed in detail. Should the patient accept respective impairments (e.g. vision loss) to achieve local tumor control, written documentation must be obtained before reirradiation. Treatment-related toxicities that occur as a consequence will be evaluated separately.

### Study endpoints

The feasibility/safety of reCIRT in patients with rHNC is the primary endpoint of the trial. The primary target value is to generate less than 35% acute/subacute severe toxicity (≥grade III) associated with study treatment within 6 months after reirradiation, according to Common Terminology Criteria for Adverse Events (CTCAE) v5.0. The local and distant progression-free survival 12 months after reirradiation, the OS, and the QoL are the secondary endpoints of the trial. Explorative trial objectives are the longitudinal investigation of clinical patient-related parameters, tumor parameters on radiological imaging, and blood-based tumor analytics.

### Study visits and evaluation criteria

**Fig. 2:**
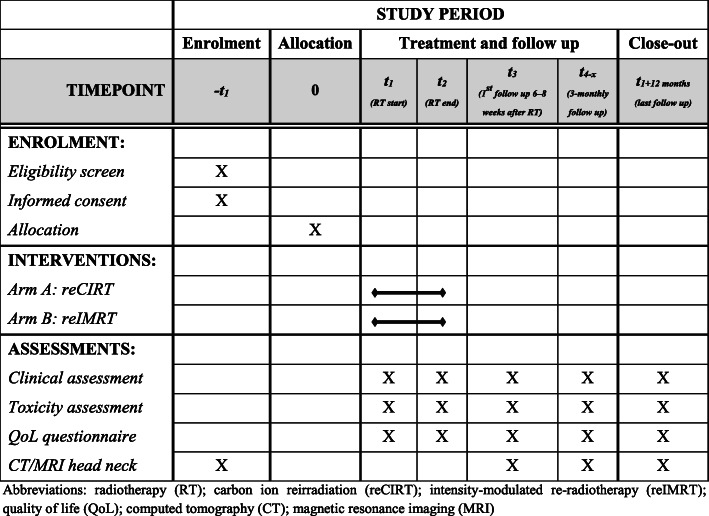
Schedule of enrolment, interventions, and assessments for the prospective randomized controlled CARE trial (SPIRIT figure)

The follow-up corresponds to the clinical routine and is not study-specific, with the exception of the QoL assessments. The first study visit will take place 6 weeks after reirradiation and thereafter every 3 months within the first year after reirradiation. Treatment response and progression will be defined according to the most recent Response Evaluation Criteria in Solid Tumors (RECIST) 1.1 [18, 19]. At the time of relapse after reirradiation, routine histological confirmation will be performed if clinically indicated (not study-specific). Detailed information on the study visits and evaluation criteria are shown in Fig. 2.

Patients will be followed-up for at least 12 months after reirradiation to document any acute and subacute CTCAE v5.0 toxicity that is related to study treatment. Patients with a partial follow-up are weighted by the proportion of the follow-up time that is completed. Acute toxicities are defined by the occurrence within the first 90 days after the start of reirradiation. Adverse events occurring after the first 90 days until 6 months after study treatment will be documented as subacute toxicities. Any adverse event emerging more than 6 months after reirradiation will be recorded as a late toxicity. Questionnaires used for QoL assessment will be the European Organisation for Research and Treatment of Cancer (EORTC) Quality of Life Questionnaire (QLQ)-C30 [20] and EORTC QLQ-H&N35 [21].

### Data management and statistics

The data are collected, managed, and processed electronically in the in-house research database. Statistical analysis is based on the International Conference on Harmonization Guidelines “Structure and Content of Clinical Study Reports” and “Statistical Principles for Clinical Trials”.

#### Power calculation

Assuming a toxicity rate for the carbon ion arm of π_Tox_^CI^ = 0.35 under the null hypothesis, and a toxicity rate of π_Tox_^CI^ = 0.15 under the alternative hypothesis, it will be possible to reject H^0^: π_Tox_^CI^ ≥ 0.35 with a probability of 1-β = 0.803 with the planned sample size of *n* = 36 patients per arm, taking a potential dropout rate of 10% into account. Furthermore, assuming a toxicity rate of π_Tox_^Ph^ = 0.35 in the photon arm and π_Tox_^CI^ = 0.15 in the carbon ion arm, a difference between treatment arms can only be demonstrated with the planned sample size and a power of 1-β = 0.8 using a chi-squared test at the one-sided significance level of α = 0.15. A comparison at a one-sided significance level of α = 0.05 would have required a total of *n* = 146 patients (including dropouts), which was deemed to be an unfeasibly high number. The power calculation was done using SAS v9.4 (SAS Institute, Cary, NC, USA).

#### Analysis of the primary endpoint

The null hypothesis for the primary (safety) endpoint of the trial is defined as H^0^: π_Tox_^CI^ ≥ 0.35 (i.e., the rate of patients with an acute/subacute toxicity CTCAE v5.0 ≥ grade III in the carbon ion arm is greater than or equal to 0.35), which is tested against its alternative H^1^: π_Tox_^CI^ < 0.35 (i.e., the rate of patients with an acute/subacute toxicity CTCAE v5.0 ≥ grade III in the carbon ion arm is less than 0.35). The null hypothesis will be tested at a one-sided significance level of α = 0.05 using an exact binomial test.

Furthermore, the toxicity rate will be estimated alongside a corresponding 90% exact Clopper–Pearson confidence interval. A (descriptive) comparison of the primary endpoint between the two treatment groups will be performed using a logistic regression model adjusting for histology (HNSCC vs. others) and RT interval (≥2 years vs. < 2 years), using a (descriptive) significance level of α = 0.15 (one-sided) for the odds ratio of the treatment group. The analysis of the primary endpoint will be based on the safety population, which comprises all patients enrolled who received at least one RT fraction.

#### Analysis of secondary endpoints

For the secondary endpoint OS, the 1-year survival rates and the median per group will be tabulated together with the respective 95% confidence intervals, and a Kaplan–Meier curve will be calculated. A descriptive stratified log-rank test adjusting for histology (HNSCC vs. others) and RT interval (≥2 years vs. < 2 years) will be conducted to compare the treatment groups. The analysis of the secondary endpoints local/distant progression-free survival will be performed analogously to the analysis of the primary endpoint OS. Furthermore, exploratory subgroup analyses will be conducted to assess potential predictors of improved efficacy. The analysis of all efficacy endpoints will be primarily based on the ITT population including all randomized patients, while the PP population containing all patients without major protocol violations will be used for sensitivity analyses. Statistical analysis will be performed using SAS v9.4 or higher (SAS Institute, Cary, NC, USA).

### Ethics and safety considerations

The study protocol, patient information sheet, and declaration of informed consent were approved by the Heidelberg University Ethics Committee (S-708/2018). The clinical trial will be performed in accordance with the current version of the Declaration of Helsinki. The recommendations of Good Clinical Practice (GCP) are taken into account regarding the performance, evaluation, and documentation of this study. The regulations concerning medical confidentiality and data protection are fulfilled.

In the clinical study, ionizing radiation is used for the purpose of medical research on humans according to §23 StrlSchV (German Radiation Protection Ordinance). The study was approved by the Federal Office for Radiation Protection.

An independent Data and Safety Monitoring Board (DSMB) will monitor recruitment, reported adverse events and data quality at least once a year. Based on its report, the DSMB will make recommendations to the Principal Investigator (PI) regarding the continuation, modification, or termination of the trial. Adverse events will be monitored and documented according to GCP guidelines.

## Discussion

The primary aim of the current randomized controlled trial is to investigate the toxicity profile of reCIRT compared to reIMRT in patients with rHNC. Previous retrospective studies on reCIRT reported encouraging results regarding safety and toxicity [14, 22]. Compared to reIMRT, the inverted dose profile and the sharp dose gradients of heavy ions allow optimal sparing of normal tissue and organs at risk [23, 24]. Therefore, the maximum allowed BED2Gy is 75 Gy for reCIRT and 60 Gy for reIMRT, limited by the dose constraints of organs at risk. We hypothesize that by using reCIRT, the cumulative rate of severe treatment-associated side effects can be significantly reduced, compared to reIMRT. The physical advantages of carbon ions are yet to be prospectively correlated with clinical toxicity profiles to validate these findings in patients with rHNC. Since many uncertainties regarding correlations of treatment characteristics with toxicity rates in reirradiation of rHNC remain, the scientific approach by itself influences the results and conclusions. Recently, the actual overlapping retreatment volume was identified as an indicator for acute treatment toxicity, but not the size of the PTV at reirradiation [25]. It is also unclear whether the point maximum or mean cumulative dose are more relevant for causing side effects and how these factors differ for different organs at risk. When treating patients with rHNC with reIMRT, due to a larger low-dose overlap, the definition of the actual retreatment volume itself is to some point arbitrary. To answer these questions, the original treatment plans therefore are required electronically for comparison or should be reconstructed on the retreatment planning CT. Based on these findings, more useful reirradiation categories, depending among others on the treatment volume overlap, can be defined, which would enhance the comparability of clinical trials. By potentially reducing treatment related toxicity, the patients’ QoL could be improved, which will be evaluated with standardized questionnaires in the current trial.

In addition, the study aims to analyze the efficacy of reCIRT compared to reIMRT in patients with rHNC. We hypothesize that reCIRT represents an effective alternative to palliative systemic therapies regarding clinical outcome parameters. Because of physiological advantages of reCIRT, enabling further dose escalation compared to reIMRT, improved local disease control and survival for patients with rHNC are conceivable [26]. Effective dose calculation in reCIRT is conducted with assumptions for different tissue types and models, e.g. the local effect model [13]. However, the relative biological effectiveness model prediction performance and correlations with patient treatment plans are subject to substantial variability [27]. Therefore, more effective dose calculations models for carbon ions combined with verification by clinical outcome evaluation with sufficiently large patient cohorts are required. Nonetheless, reCIRT may overcome radioresistance due to the increased RBE, e.g. by eradicating tumor stem cells [28]. Identifying patients that benefit significantly from reCIRT represents a major challenge, since a multitude of factors are relevant. A predictive score for reirradiation could possibly guide clinical decision-making, if for this purpose relevant patient and treatment factors are identified from randomized controlled trials. In the current trial, clinical outcome parameters will be correlated with blood-based analytics and tumor parameters on radiological imaging to further explore and validate our findings. At present, this investigation is the first randomized controlled trial prospectively comparing reCIRT to reIMRT in patients with rHNC.

## Data Availability

Not applicable.

## Abbreviations

reIMRT: intensity-modulated re-radiotherapy
rHNC: recurrent head and neck cancer
reCIRT: carbon ion reirradiation
OS: Overall survival
QoL: Quality of life
RBE: Relative biological effectiveness
HNC: Head and neck cancer
HNSCC: Head and neck squamous cell carcinoma
SBRT: Stereotactic body radiation therapy
RT: Radiotherapy
CT: Computed tomography
MRI: Magnetic resonance imaging
CTV: Clinical target volume
PTV: Planning target volume
GTV: Gross tumor volume
LEM: Local effect model
BED2Gy: Biological effective dose in 2 Gray fractions
CTCAE: Common Terminology Criteria for Adverse Events
EORTC: European Organisation for Research and Treatment of Cancer
DSMB: Data and Safety Monitoring Board
